# *Toxoplasma Gondii* Moderates the Association between Multiple Folate-Cycle Factors and Cognitive Function in U.S. Adults

**DOI:** 10.3390/nu9060564

**Published:** 2017-06-02

**Authors:** Andrew N. Berrett, Shawn D. Gale, Lance D. Erickson, Bruce L. Brown, Dawson W. Hedges

**Affiliations:** 1 Department of Psychology, Brigham Young University, Provo, UT 84602, USA; shawn_gale@byu.edu (S.D.G.); bruce_brown@byu.edu (B.L.B.); dawson_hedges@byu.edu (D.W.H.); 2 The Neuroscience Center, Brigham Young University, Provo, UT 84602, USA; 3 Department of Sociology, Brigham Young University, PProvo, UT 84602, USA; lance_erickson@byu.edu

**Keywords:** *Toxoplasma gondii*, Folate, Cognition, Memory, Vitamin B-12, Homocysteine

## Abstract

*Toxoplasma gondii* (*T. gondii*) is a microscopic, apicomplexan parasite that can infect muscle or neural tissue, including the brain, in humans. While *T. gondii* infection has been associated with changes in mood, behavior, and cognition, the mechanism remains unclear. Recent evidence suggests that *T. gondii* may harvest folate from host neural cells. Reduced folate availability is associated with an increased risk of neurodevelopmental disorders, neurodegenerative diseases, and cognitive decline. We hypothesized that impairment in cognitive functioning in subjects seropositive for *T. gondii* might be associated with a reduction of folate availability in neural cells. We analyzed data from the third National Health and Nutrition Examination Survey to determine the associations between *T. gondii* infection, multiple folate-cycle factors, and three tests of cognitive functioning in U.S. adults aged 20 to 59 years. In these analyses, *T. gondii* moderated the associations of folate, vitamin B-12, and homocysteine with performance on the Serial Digit Learning task, a measure of learning and memory, as well as the association of folate with reaction time. The results of this study suggest that *T. gondii* might affect brain levels of folate and/or vitamin B-12 enough to affect cognitive functioning.

## 1. Introduction

*Toxoplasma gondii* (*T. gondii*) is an apicomplexan parasite that infects approximately 12% of the U.S. population as of 2010 [1]. Although the parasite’s definitive host is any member of the cat family, humans can become infected with *T. gondii* as an intermediate host via ingestion of contaminated foods or by exposure to cat feces [2]. Upon successful invasion, *T. gondii* infects muscle and neural tissue and encapsulates itself in a cyst that protects it against the host immune system [3]. *T. gondii* biology has unique features that set it apart from other intracellular parasites. For example, using precursors from the infected cell, *T. gondii* can synthesize dopamine [4,5], although the physiological effects of this dopamine production remain unknown.

Beyond dopamine production, *T. gondii* may influence other aspects of host biology. In regards to folate synthesis, *T. gondii* may salvage or harvest folate directly from the infected host cell [6]. It is unknown, however, whether this might affect host health. Further, it is unclear whether *T. gondii* depends on the folate acquired from the host or if the system is only supplementary.

In humans, folate is essential for DNA synthesis and repair and is a key factor in cell division and growth [7]. The brain requires a constant supply of dietary folate for early embryonic neurodevelopment, adult neurogenesis, and the production of neurotransmitters [8,9]. Therefore, reduced availability of folate is associated with neurodevelopmental disorders [10] and may be associated with reduced cognitive function [11,12,13,14].

In individuals who do not obtain sufficient dietary folate or who cannot metabolize folate efficiently, neural cells might be generally and persistently deprived of this essential nutrient [15,16]. If *T. gondii* is indeed capable of harvesting folate from host neural cells, then infected cells may be further starved of this important nutrient and thereby magnify potential impairments in cognitive functioning. Based on these factors, we hypothesized that cognitive function may be associated with an interaction between *T. gondii* infection and concentrations of folate and other factors related to folate metabolism such as vitamin B-12 and homocysteine. To test this, we used the National Health and Nutrition Examination Survey III (NHANES III) dataset from the United States’ Centers for Disease Control and Prevention (CDC).

## 2. Materials and Methods

### 2.1. Study Sample

**Figure 1:**
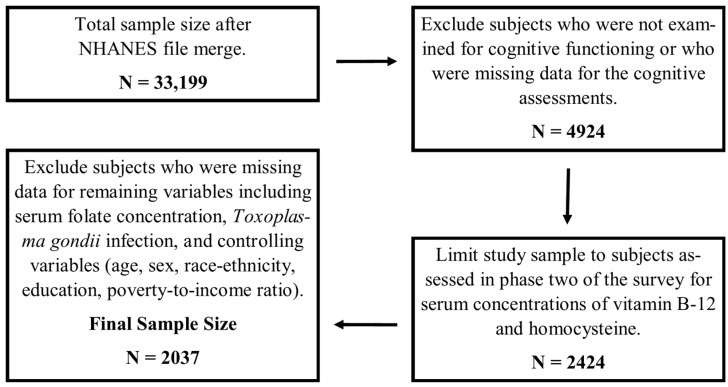
Flow diagram of sampling selection and final number of subjects included in all analyses.

The NHANES III is a nationally representative survey conducted by the CDC from 1988 to 1994. It includes demographic, examination, laboratory, and dietary information for approximately 34,000 participants recruited from multiple locations around the U.S. Using stratified sampling methods and statistical weighting, the NHANES III was designed to represent the non-institutionalized U.S. population. In the NHANES III, a subsample of subjects aged 20 to 59 years completed tests of cognitive functioning thereby limiting the potential study sample to 4924. Data for serum concentrations of vitamin B-12 and homocysteine were only collected in subjects who were assessed during the second phase (1991–1994) of the NHANES survey, further limiting the potential study sample to 2242 subjects. Finally, we did not include in our analyses subjects who were missing data for any of the remaining variables we used in this study (i.e., serum folate concentration, *T. gondii* infection status, and all controlling variables), resulting in a final sample size of 2037 (Figure 1).

### 2.2. Toxoplasma Gondii

Detection of *T. gondii*-specific antibodies was accomplished by CDC laboratory technicians via indirect enzyme immunoassay. A full description of the laboratory procedures used is available in the NHANES III laboratory manual [17]. Briefly, test samples were combined with *T. gondii* antigens, peroxidase-labelled human gamma chain immunoglobulin (IgG), peroxidase substrate, and chromogen ortho-phenylene diamine resulting in a color change proportional to the total *T. gondii* antibody concentration in the sample. The color change is read as an optical density via spectrophotometer and compared to a standard curve calibrated to World Health Organization Toxo 60 serum. Possible IgG titer values ranged from 0 IU/mL to 240 IU/mL (higher concentrations were collapsed to 240 IU/mL to reduce the risk of deductive disclosure). Subjects with titer values above 7 IU/mL were considered seropositive for *T. gondii* exposure, whereas subjects below this level were considered negative [17].

Anti-*T. gondii* IgG antibodies indicate past exposure to *T. gondii*. Further, IgG titer levels can vary across the lifespan potentially due to multiple exposures or natural anamnestic decreases in IgG levels [18,19,20]. However, the procedures and classification system used in the NHANES III remain the industry norm (excepting minor differences in the IgG titer cut-off point for seropositivity depending on the specific assay/manufacturer) in identifying individuals exposed to and likely infected with *T. gondii*.

### 2.3. B-Vitamin Biomarkers

Serum concentrations of folate and vitamin B-12 were determined by CDC laboratory technicians using a Quantaphase Folate radioassay kit from Bio-Rad Laboratories. The Quantaphase Folate radioassay kit contains a detailed description of the methods used to prepare the folate and B-12 samples [21]. Following sample preparation, concentration is calculated from a standard curve. Serum homocysteine concentration was determined via reverse-phase, high-performance liquid chromatography and fluorescence detection as described in the NHANES III laboratory manual [17]. Importantly, data for the NHANES III was collected before the introduction of mandatory folic acid food fortification [22]. Therefore, reported average serum concentrations of folate and/or vitamin B-12 for subjects surveyed in the NHANES III may be lower than more recent averages.

### 2.4. Cognitive Functioning

In the NHANES III, computerized versions of the Symbol Digit Substitution (SDS), Serial Digit Learning (SDL), and Reaction Time (RT) tests assessed general cognitive function. In the SDS, subjects were required to match a series of symbols with their respective digits using a key given at the top of the computer screen for four trials. Each trial consisted of nine digit-symbol pairings, and the total latency (in seconds) for each of the nine possible symbol-digit pairings was recorded for each trial. Finally, the average of the two lowest total latencies from the four trials was used as the final score. Therefore, a lower score, or faster pairing, on the SDS indicates better performance. The SDL required subjects to memorize and recall a series of eight random digits over eight trials. Scores on the SDL reflected the number of errors made in each trial. Therefore, a lower score, or fewer errors, indicates better performance on the SDL. Subjects who successfully repeated the eight-digit sequence for two consecutive trials were not required to complete the remaining trials. Finally, RT was measured by pressing a button as quickly as possible whenever a square appeared in the center of the computer screen. The average RT (in milliseconds (ms)) over 50 trials was used as the final score with faster times indicating better RT performance. Krieg et al. [23] provides a detailed description of the three cognitive tests used in the NHANES III.

### 2.5. Covariates

We included a number of covariates in all analyses to control for potential confounding. Continuous covariates included age in years, poverty-to-income ratio (PIR), and years of education. PIR is a measure of an individual’s socioeconomic status computed by dividing the household income by the federal poverty level (at the time of the survey) resulting in a minimum value of zero and a maximum of five (higher values were collapsed to five to reduce the risk of deductive disclosure). Categorical variables included sex and race-ethnicity. For the race-ethnicity variable, the NHANES III survey asked all participants to self-report one of the following race-ethnicity categories: non-Hispanic white, non-Hispanic black, Mexican American, or “other”.

### 2.6. Statistical Analysis

We used Stata 14.2 [24] for all statistical analyses. We computed summary statistics for all variables included in our analyses and computed means and standard errors for continuous variables and proportions and standard errors for categorical variables. Ordinary least squares regression was used to test for differences between *T. gondii* seropositive and seronegative subjects on each of the variables included in this study. Only the *p*-values for these comparisons are reported in Table 1. We used ordinary least squares regression to test for interaction effects between *T. gondii* seropositivity and folate, vitamin B-12, or homocysteine concentrations in the prediction of SDS, SDL, or RT scores for a total of nine separate models. We included age, PIR, education, sex, and race-ethnicity as covariates in each model.

For each analysis, we report main effects for *T. gondii*, a folate-cycle factor, and their interaction. The coefficient for *T. gondii* is the score on the respective cognitive test for subjects seropositive for *T. gondii* infection. The coefficient for the folate-cycle factors represents the relationship between each factor and scores on each of the three cognitive tests for subjects who were seronegative for *T. gondii* infection. The interaction of folate-cycle factors and *T. gondii* is the change in the slope of the folate-cycle factor for those subjects seropositive for *T. gondii* relative to those seronegative for *T. gondii*.

## 3. Results

Of the 2037 subjects included in this study, approximately 20% were seropositive for *T. gondii*, 50% were female, and approximately 75% were non-Hispanic white. Table 1 presents these and other demographic and study-sample characteristics.

**Figure 2:**
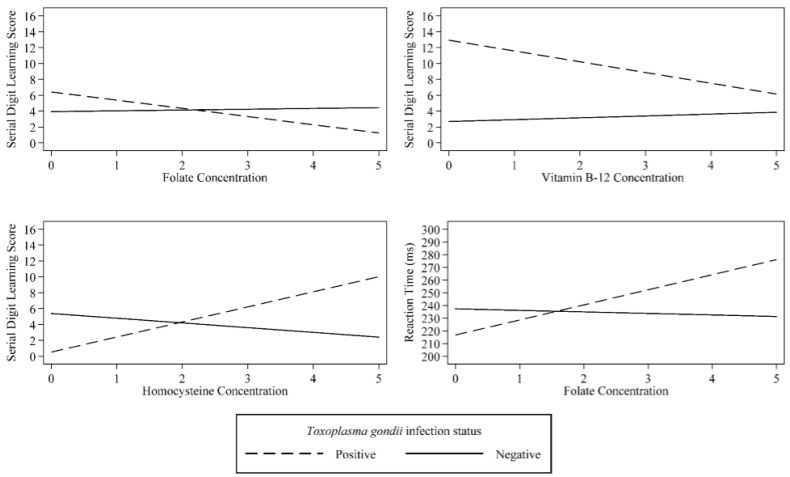
Significant interaction between folate-cycle factors and *Toxoplasma gondii* predicting cognitive performance in U.S. adults. **Notes:** Higher values on each cognitive assessment indicate worse performance. Values for folate, vitamin B-12, and homocysteine were logarithmically transformed.

We found no significant interactions between *T. gondii* seropositivity and any of the folate-cycle factors in predicting performance on the SDS. In predicting performance on the SDL, *T. gondii* seropositivity interacted with folate (β = −1.02, (95% CI: −1.88, –0.16), *p* = 0.021; Table 2), vitamin B-12 (β = −1.60, (95% CI: −2.98, −0.23), *p* = 0.023; Table 3), and homocysteine concentrations (β = 2.40, (95% CI: 0.94, 3.85), *p* = 0.002; Table 4) (Figure 2). In subjects seronegative for *T. gondii*, SDL performance was relatively constant as folate, vitamin B-12 or homocysteine levels varied. However, in subjects seropositive for *T. gondii*, performance on the SDL worsened as folate and vitamin B-12 levels decreased and as homocysteine levels increased (Figure 2). An interaction between *T. gondii* seropositivity and folate concentration predicted RT (β = 14.88, (95% CI: 5.99, 23.76), *p* = 0.001; Table 2). In contrast to the SDL, subjects seropositive for *T. gondii* performed better on the RT assessment as folate concentration decreased.

## 4. Discussion and Conclusions

Using a large, nationally representative sample of U.S. adults, this study provides evidence of an interaction between *T. gondii* seropositivity and concentrations of several folate-cycle factors in the prediction of general cognitive functioning. In this sample of over 2000 participants, approximately 20% were seropositive for IgG antibodies specific for *T. gondii*. While this prevalence of seropositivity is higher than more recent estimates [1], it is within the expected range for the years in which the NHANES III was conducted (1988–1994) [25,26].

In this study, interactions between *T. gondii* seropositivity and folate, vitamin B-12, and homocysteine concentrations particularly affected performance on the SDL, an assessment of ability in learning and recall [23] that requires attention and intact short-term memory to perform well on the task. Concentrations of folate-cycle factors (including folate, vitamin B-12, and homocysteine) have been found to be associated with memory loss and other cognitive impairments in neurological disorders such as dementia [12,13,27], although these studies did not take into account *T. gondii* seropositivity. Further, deficiency in dietary folate has been linked to reduced genesis of neuroprogenitor cells of the adult hippocampus [8], a brain region that influences both short-term memory and attention [28,29]. Low concentrations of folate and/or vitamin B-12 usually result in an elevation of homocysteine, which can damage neural cells and impair cognitive functioning. Indeed, elevated homocysteine also might increase risk for neurodegenerative disorders in the hippocampus and prefrontal cortex [12,30]. Therefore, reductions in cellular folate concentrations, such as in the case of folate harvesting by *T. gondii*, could limit the bioavailability of this important nutrient and cause elevations in homocysteine in multiple brain regions including the prefrontal cortex or hippocampus [31,32] and thus disrupt learning, attention, or other related cognitive functions. Further, this effect may be magnified in individuals who do not regularly obtain sufficient amounts of dietary folate or who cannot metabolize folate efficiently.

Another potential mechanism by which reduced folate availability might impact cognitive functioning lies in the indirect role of folate metabolism in the generation of serotonin and dopamine. Purines synthesized during folate metabolism contribute to the biosynthesis of guanosine triphosphate (GTP), a precursor of tetrahydrobiopterin (BH_4_). Tyrosine hydroxylase and tryptophan hydroxylase each utilize BH_4_ as a co-factor in the production of dopamine and serotonin, respectively. Therefore, reduced availability of dietary folate may indirectly lead to an overall decrease of dopamine and serotonin production [9,33]. In the case of *T. gondii* seropositivity, the combined effects of low concentrations of folate and simultaneous folate harvesting from *T. gondii* might particularly affect infected neural cells. Since dopamine and serotonin both influence various cognitive functions such as memory, attention, and executive function [34,35,36], this link could potentially offer an alternate explanation of the effects observed in this study.

Beyond performance on the SDL, there was a significant interaction between *T. gondii* and folate concentration in the prediction of RT. However, the direction of the observed relationship suggested that *T. gondii* seropositivity might actually be favorable in regards to RT in cases of low folate concentration and detrimental in cases of high folate concentration. Though not statistically significant, we found a similar relationship in a previous study [37] exploring interactions between the bacterium *Helicobacter pylori* and folate concentration in the prediction of RT. Similar to *T. gondii*, *Helicobacter pylori* might also reduce folate availability [38], though by a different mechanism. Despite these differing mechanisms of folate reduction, in both cases, RT improved, suggesting that folate deficiency associated with *T. gondii* might enable some advantage in RT. We are unaware of a mechanism that might explain how reductions in folate availability by infectious diseases such as *T. gondii* or *Helicobacter pylori* could be favorable towards RT performance. The data available in the NHANES III do not allow for an in-depth exploration of this association. Future research into this finding would likely require animal models or neuroimaging combined with biochemical testing to determine how regions of the brain active during RT tasks might be affected by reductions in folate availability.

Some limitations require consideration when interpreting the results of this study. The NHANES data sets are cross-sectional in design and thus preclude the ability to make casual inferences. Further, as the degree to which *T. gondii* harvests folate from infected host cells is not clear, it is currently unknown whether *T. gondii* harvests enough to affect cognitive functioning. With these data, it is also impossible to determine the time of initial infection by *T. gondii* or the frequency with which individuals have encountered the parasite in their lifetime. It is possible that the effects of *T. gondii* on folate availability and on cognition could vary based on the length of infection with the parasite or based on the number of exposures to the parasite. Finally, folate and vitamin B-12 availability can vary for a multitude of reasons including genetic mutations in key folate-cycle enzymes [7,39,40]. Although the NHANES III does include genetic data, access to the data is restricted and requires funding to obtain it. Therefore, additional research is needed to identify other factors that might modify the effects observed in this study.

This study is strengthened by using multiple controls that limit the number of potential confounds that might explain the observed effects. Specifically, by controlling for demographic and other health factors, we were more confident that the interactions between *T. gondii* and concentrations of folate, vitamin B-12, or homocysteine were associated with the variation in SDL or RT scores. Finally, use of the NHANES III data sets resulted in a large sample size that increases the generalizability of the findings and study power.

This study consisting of 2037 U.S. adults presents evidence of an interaction effect between *T. gondii* seropositivity and concentrations of multiple folate-cycle factors on cognitive functioning in adult humans. Additional research is recommended to explore the specific mechanisms involved in this association and to determine any additional potential consequences to folate and/or vitamin B-12 availability following *T. gondii* infection.

## Figures and Tables

**Table 1:** Weighted summary statistics of U.S. adults 20 to 59 year olds, National Health and Nutrition Examination Survey III 1988–1994.

	*T. Gondii* Seropositive *N* = 417			*T. Gondii* Seronegative *N* = 1620	
	Mean or Frequency	SE	Mean or Frequency	SE	*p*
Sociodemographic Factors					
Age (years)	41.54	1.01	36.16	0.63	<0.001
Poverty-to-income Ratio	3.23	0.25	3.38	0.13	0.316
Education (years)	12.36	0.23	13.13	0.12	<0.001
Female	45.63%	2.65%	51.55%	1.08%	0.050
Race-ethnicity					
Non-Hispanic White	72.73%	3.92%	77.98%	1.27%	0.159
Non-Hispanic Black	10.77%	1.64%	10.55%	0.47%	0.896
Hispanic	5.87%	0.81%	5.50%	0.40%	0.691
Other	10.62%	3.25%	5.97%	1.14%	0.097
Biochemistry					
Folate (ng/mL)	7.08	0.51	6.89	0.24	0.807
Vitamin B-12 (pg/mL)	521.83	44.25	474.01	7.08	0.623
Homocysteine (umol/L)	9.36	0.27	9.56	0.25	0.282
Cognitive Testing					
Symbol Digit Substitution Score	2.85	0.05	2.55	0.03	<0.001
Serial Digit Learning Score	5.45	0.31	3.91	0.15	<0.001
Reaction Time (sec)	240.1	3.03	234.47	1.71	0.070

Abbreviations: SE = Standard Error.

**Table 2:** Two-way interaction effects of serum folate concentration and *Toxoplasma gondii* infection status predicting SDS, SDL, and RT cognitive assessment scores for U.S. 20 to 59 year olds.

	Symbol Digit Substitution			Serial Digit Learning			Reaction Time		
	β	95% CI	*p*	β	95% CI	*p*	β	95% CI	*p*
Folate	0.01	(−0.04, 0.05)	0.786	0.10	(−0.33, 0.53)	0.641	−1.19	(−5.55, 3.16)	0.585
*T. gondii*	0.15	(−0.08, 0.38)	0.185	2.48	(1.02, 3.94)	0.001	−20.60	(−34.38, −6.83)	0.004
*T. gondii* × Folate Interaction	−0.07	(−0.20, 0.07)	0.344	−1.13	(−1.97, −0.29)	0.009	13.07	(4.20, 21.93)	0.005
Race-Ethnicity									
Non-Hispanic White (ref)	-	-	-	-	-	-	-	-	-
Non-Hispanic Black	0.30	(0.21, 0.39)	<0.001	1.72	(1.15, 2.29)	<0.001	5.92	(0.14, 11.70)	0.045
Mexican American	0.27	(0.18, 0.37)	<0.001	2.52	(1.95, 3.09)	<0.001	1.23	(−6.86, 9.32)	0.761
Other	0.29	(0.10, 0.47)	0.003	1.88	(0.69, 3.08)	0.003	−4.15	(−14.36, 6.06)	0.418
Female	−0.17	(−0.24, −0.10)	<0.001	0.08	(−0.23, 0.38)	0.611	8.63	(1.60, 15.66)	0.017
Age (years)	0.03	(0.03, 0.03)	<0.001	0.09	(0.07, 0.11)	<0.001	0.36	(0.14, 0.58)	0.002
PIR	−0.03	(−0.04, −0.02)	<0.001	−0.25	(−0.35, −0.14)	<0.001	−3.13	(−4.33, −1.92)	<0.001
Education (years)	−0.10	(−0.11, −0.08)	<0.001	−0.46	(−0.57, −0.35)	<0.001	−2.93	(−4.08, −1.79)	<0.001
Constant	2.91	(2.70, 3.12)	<0.001	6.77	(4.96, 8.57)	<0.001	267.68	(250.01, 285.34)	<0.001

Notes: Age, sex, education, race-ethnicity, and poverty-to-income ratio included as controls in all models. Abbreviations: SDS, the Symbol Digit Substitution; SDL, Serial Digit Learning; RT, Reaction Time; CI = Confidence Interval; ref = Reference category; PIR, poverty-to-income ratio. *N* = 2037.

**Table 3:** Two-way interaction effects of serum vitamin B-12 concentration and *Toxoplasma gondii* infection status predicting SDS, SDL, and RT cognitive assessment scores for U.S. 20 to 59 year olds.

	Symbol Digit Substitution			Serial Digit Learning			Reaction Time		
	β	95% CI	*p*	β	95% CI	*p*	β	95% CI	*p*
Vitamin B-12	0.05	(−0.00, 0.11)	0.057	0.23	(−0.26, 0.72)	0.346	−3.04	(−8.77, 2.68)	0.291
*T. gondii*	1.06	(−0.12, 2.25)	0.076	10.24	(2.14, 18.33)	0.014	5.26	(−123.23, 133.74)	0.935
*T. gondii* Vitamin B-12 Interaction	−0.17	(−0.36, 0.03)	0.087	−1.59	(−2.92, −0.26)	0.020	−0.61	(−21.40, 20.19)	0.954
Race-Ethnicity									
Non-Hispanic White (ref)	-	-	-	-	-	-	-	-	-
Non-Hispanic Black	0.30	(0.21, 0.39)	<0.001	1.78	(1.19, 2.37)	<0.001	6.09	(0.18, 12.01)	0.044
Mexican American	0.27	(0.18, 0.37)	<0.001	2.53	(1.96, 3.10)	<0.001	1.81	(−6.22, 9.84)	0.653
Other	0.29	(0.10, 0.47)	0.003	1.87	(0.67, 3.08)	0.003	−4.59	(−14.98, 5.81)	0.380
Female	−0.17	(−0.24, −0.10)	<0.001	0.08	(−0.21, 0.36)	0.583	8.52	(1.64, 15.40)	0.016
Age (years)	0.03	(0.03, 0.03)	<0.001	0.09	(0.07, 0.11)	<0.001	0.40	(0.17, 0.63)	0.001
PIR	−0.03	(−004, −0.02)	<0.001	−0.24	(−0.34, −0.14)	<0.001	−3.14	(−4.32, −1.97)	<0.001
Education (years)	−0.10	(−0.11, −0.08)	<0.001	−0.47	(−0.58, −0.35)	<0.001	−2.83	(−4.03, −1.64)	<0.001
Constant	2.61	(2.22, 2.99)	<0.001	5.68	(2.42, 8.93)	0.001	281.51	(239.25, 323.77)	<0.001

Notes: Age, sex, education, race-ethnicity, and poverty-to-income ratio included as controls in all models. Abbreviations: CI = Confidence Interval, ref = Reference category. *N* = 2037.

**Table 4:** Two-way interaction effects of serum homocysteine concentration and *Toxoplasma gondii* infection status predicting SDS, SDL, and RT cognitive assessment scores for U.S. 20 to 59 year olds.

	Symbol Digit Substitution			Serial Digit Learning			Reaction Time		
	β	95% CI	*p*	β	95% CI	*p*	β	95% CI	*p*
Homocysteine	−0.05	(−0.13, 0.02)	0.166	−0.59	(−1.22, 0.03)	0.060	−2.72	(−9.05, 3.62)	0.393
*T. gondii*	−0.35	(−1.17, 0.48)	0.403	−4.87	(−7.88, −1.85)	0.002	−10.87	(−45.43, 23.70)	0.531
*T. gondii* × Homocysteine Interaction	0.18	(−0.20, 0.56)	0.351	2.49	(1.09, 3.89)	0.001	5.67	(−11.10, 22.43)	0.501
Race-Ethnicity									
Non-Hispanic White (ref)	-	-	-	-	-	-	-	-	-
Non-Hispanic Black	0.31	(0.22, 0.40)	<0.001	1.79	(1.19, 2.38)	<0.001	5.50	(−0.33, 11.34)	0.064
Mexican American	0.27	(0.17, 0.37)	<0.001	2.52	(1.96, 3.08)	<0.001	1.41	(−6.81, 9.62)	0.732
Other	0.29	(0.11, 0.48)	0.002	1.98	(0.81, 3.15)	0.001	−3.92	(−13.95, 6.11)	0.436
Female	−0.18	(−0.25, −0.10)	<0.001	0.02	(−0.31, 0.34)	0.926	8.13	(1.12, 15.13)	0.024
Age (years)	0.03	(0.03, 0.03)	<0.001	0.09	(0.07, 0.11)	<0.001	0.42	(0.18, 0.66)	0.001
PIR	-0.03	(−0.05, −0.02)	<0.001	−0.25	(−0.35, −0.15)	<0.001	−3.21	(−4.45, −1.98)	<0.001
Education (years)	-0.10	(−0.11, −0.08)	<0.001	−0.47	(−0.59, −0.36)	<0.001	−2.83	(−4.02, −1.64)	<0.001
Constant	3.04	(2.78, 3.30)	<0.001	8.36	(6.08, 10.65)	<0.001	268.43	(243.08, 293.78)	<0.001

Notes: Age, sex, education, race-ethnicity, and poverty-to-income ratio included as controls in all models. Abbreviations: CI = Confidence Interval, ref = Reference category. *N* = 2037.
